# Taxonomic notes on the afrotropical genera *Hapalogenius* Hagedorn, *Hylesinopsis* Eggers, and *Rhopalopselion* Hagedorn (Coleoptera, Curculionidae, Scolytinae)

**DOI:** 10.3897/zookeys.56.523

**Published:** 2010-09-17

**Authors:** Roger A Beaver

**Affiliations:** 161/2 Mu 5, Soi Wat Pranon, T. Donkaew, A. Maerim, Chiangmai 50180, Thailand

**Keywords:** Afrotropical region, Curculionidae, Hapalogenius, Hylesinopsis, Rhopalopselion, Scolytinae, new synonymy, new combination

## Abstract

Taxonomic confusion among the afrotropical scolytine genera Hapalogenius Hagedorn, Hylesinopsis Eggers and Rhopalopselion Hagedorn, and their synonyms is discussed with especial reference to the catalogues of Wood and Bright (1992), and Alonso-Zarazaga and Lyal (2009). A key is given to separate the three genera recognised, and the species considered to be included in each genus are listed. Hylesinopsis is resurrected from synonymy with Hapalogenius, and shown not to be closely related to it. Chilodendron Schedl is considered to be a synonym of Hylesinopsis and not of Xylechinus Chapuis. The following new synonymy is proposed at specific level: Hapalogenius africanus (Eggers) (= Hapalogenius lesnei Eggers, = Metahylesinus brincki Schedl); Hapalogenius fuscipennis (Chapuis) (= Hapalogenius bimaculatus Eggers); Hapalogenius oblongus (Eggers) (= Metahylesinus striatus Schedl); Hylesinopsis fasciata (Hagedorn) (= Kissophagus punctatus Eggers); Phrixosoma niger Eggers (= Hapalogenius niger Schedl). The following species are returned to Hylesinopsis from Hapalogenius to which they were transferred by Alonso-Zarazaga and Lyal (2009): Hylesinopsis alluaudi (Lepesme), Hylesinopsis angolensis (Schedl), Hylesinopsis arabiae (Schedl), Hylesinopsis atra (Nunberg), Hylesinopsis confusa (Eggers), Hylesinopsis decellei (Nunberg), Hylesinopsis dubia Eggers, Hylesinopsis emarginata (Nunberg), Hylesinopsis fasciata (Hagedorn), Hylesinopsis ficus (Schedl), Hylesinopsis granulata (Lepesme), Hylesinopsis hirsuta (Schedl), Hylesinopsis joveri (Schedl), Hylesinopsis pauliani (Lepesme), Hylesinopsis punctata (Eggers), Hylesinopsis saudiarabiae (Schedl). The following new combination is given: Hylesinopsis leprosula (Browne) from Cryphalus Erichson. New distributional records are given for some species.

## Introduction

There has been considerable confusion in the literature about the relationships and limits of the scolytine genera Hapalogenius Hagedorn, Hylesinopsis Eggers, Rhopalopselion Hagedorn, and some other scolytine nominal genera from the Afrotropical region. In this paper, I attempt to resolve some of this confusion, and give some resultant taxonomic changes. The conclusions are based on the study of type material and other specimens from the following institutions: Deutsches Entomologisches Institut, Müncheberg (DEI), Hungarian Natural History Museum, Budapest (NHMB), Musée Royale de l’Afrique Centrale, Tervuren (MRAC), Museum and Institute of Zoology, Polish Academy of Sciences, Warsaw (MIZW), Museum fur Naturkünde der Humboldt Universität, Berlin (MNB), National Collection of Insects, Pretoria (NCIP), Natural History Museum, London (NHML), Naturhistorisches Museum, Wien (NMW), Transvaal Museum, Pretoria (TMP), Zoological Museum of Lund University (ZMLU), supplemented by specimens sent for identification by B. Jordal (University of Bergen, Norway), and in my own collection (RAB).

The genus Rhopalopselion was described by Hagedorn (1909) with Rhopalopselion bituberculatum Hagedorn the only included species. The genus Hapalogenius was described by Hagedorn (1912) with Hapalogenius globosus the only included species. The genus Hylesinopsis was described by Eggers (1920b) with Hylesinopsis dubius the only included species. Schedl (1951) considered that Rhopalopselion and Hapalogenius (together with a third genus, Pseudophloeotribus Eggers) were synonymous, an opinion that he later retracted (Schedl 1963a). In fact, Schedl (1963a) placed Rhopalopselion, Hapalogenius and Hylesinopsis in three different tribes, Strombophorini, Hypoborini and Phloeosini [sic] respectively, within his subfamily Hylesinae [sic]. Wood (1978, 1986) continued to consider Hapalogenius as a synonym of Rhopalopselion, and included it and Hylesinopsis in the tribe Hylesinini. Between 1983 and 1988, Wood synonymised with Hylesinopsis the following genera: Trypographus Schedl, Chilodendron Schedl (Wood 1983); Metahylesinus Eggers (a replacement name for Pseudohylesinus Eggers nec Swaine), Hapalophloeus Schedl, Hemihylesinus Schedl (Wood 1984); Glochicopterus Schedl, a genus synonymised with Metahylesinus by Wood (1983); and Aridiamerus Schedl (Wood 1988a,b). In addition, Schedl (1957a) had already synonymised Pseudophloeotribus Eggers with Metahylesinus, a synonymy accepted by Wood (1986). Wood and Bright (1992) in their catalogue of Scolytidae maintain this position, and give further references to the genera and species mentioned.

Recently, Alonso-Zarazaga and Lyal (2009) recognised that Wood and Bright (1992) had placed the type species of Hapalogenius in synonymy with Hylesinopsis fuscipennis (Chapuis), and consequently reinstated Hapalogenius as the valid name for the genus Hylesinopsis. This resulted in a large number (38) of recombinations of species transferred from Hapalogenius to Hylesinopsis. Alonso-Zarazaga and Lyal (2009) retain Rhopalopselion as a valid genus, with the same twenty-four species included by Wood and Bright (1992).

**Figure 1.**
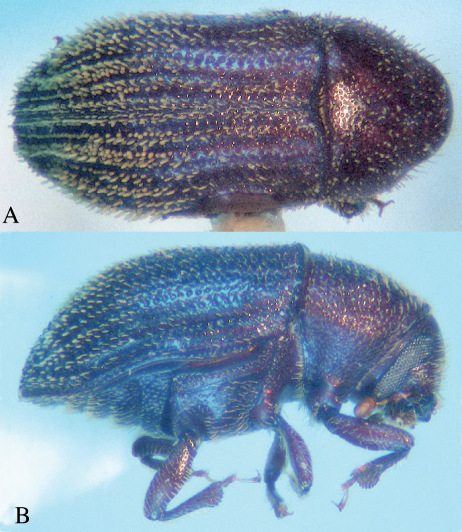
Hapalogenius oblongus (Eggers), dorsal, **A** and lateral, **B**.

**Figure 2.**
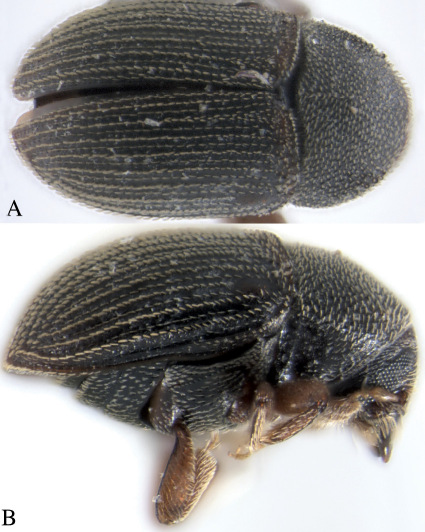
Hylesinopsis dubius Eggers, dorsal, **A** and lateral, **B**.

**Figure 3.**
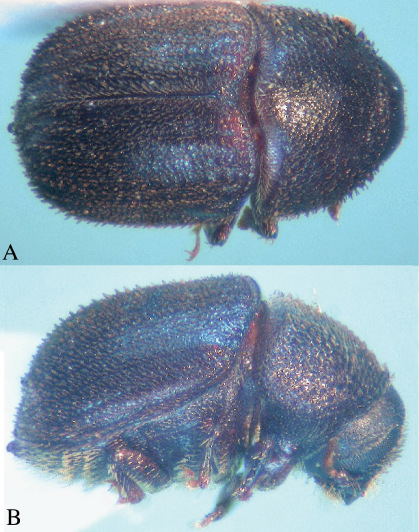
Rhopalopselion thompsoni Schedl, dorsal, **A** and lateral, **B**.

Wood (1986) has suggested that Hylesinopsis *sensu* Woodand Rhopalopselion *sensu* Wood are rapidly evolving genera, and that they could either be amalgamated into a single, large genus, or split up into a number of small genera. I believe that the most satisfactory solution at present is to distinguish three genera, based on morphological and biological criteria: Rhopalopselion, Hapalogenius and Hylesinopsis. The first two of these genera are closely related, the third, Hylesinopsis, is quite distinct from them. Figures 1–3 illustrate a representative species of each genus. The three genera may be distinguished using the following key, which also serves to diagnose the genera:

**Table:** 

1.	Antennal funicle 7-segmented, club rounded to ovate with several annuli of closely-placed hairs. Pronotum almost quadrate, without a subapical constriction, the anterior angles prominently spinulose with strong asperities. Scutellum rather large, quadrate. Fifth abdominal ventrite with a median, approximately triangular process. Protibia with closely placed socketed teeth, and a well-developed tarsal groove on the anterior side. Large or moderately sized, stoutly-built, black species, 2.5 mm or more long. Xylophagous	Rhopalopselion Hagedorn
–	Antennal funicle 6- or 7-segmented, club sometimes more elongate, with or without more rows of setae than segments. Pronotum trapezoidal, narrowed anteriorly, usually with a subapical constriction, the anterior angles less prominently or not spinulose, the asperities often weakly developed. Scutellum small or not visible. Fifth abdominal ventrite without a median process. Protibia with or without socketed teeth; tarsal groove strongly or weakly developed. Smaller, less robust, usually brown or ferruginous species, usually less than 2.5 mm long. Phloeophagous, except Hapalogenius horridus (Eggers), which is reportedly xylophagous	2
2.	Antennal club oblong-oval or egg-shaped, apex rounded, sometimes septate, usually with 4–7 annuli of closely placed hairs not corresponding to the sutures. Funicle 6- or 7-segmented, at least the last 1–2 segments much more strongly transverse than the more basal segments, symmetrically inserted into club. Eye usually shallowly emarginate. Costate ridge extends from procoxa to anteroventral margin of pronotum. Apical half of protibia widened, with convex outer margin rounded to apex and bearing 5–9 small, closely placed socketed teeth. Anterior face of protibia with well-developed, glabrous tarsal groove, anterior tarsi retractile into groove	Hapalogenius Hagedorn
–	Antennal club elongate, apex somewhat pointed, basal suture usually partly or completely septate, with no more than 3 rows of setae on outer face. Funicle always 6-segmented, the apical segments not strongly transverse, usually only slightly wider than more basal segments, more or less asymmetrically inserted into club. Eye entire. Costate ridge between procoxa and anteroventral margin of pronotum absent. Outer margin of protibia not convexly rounded in apical half, without a series of small socketed teeth, extended apically into a backwardly-pointing spine, at most 3 smaller spines basal to it on the outer margin, apical margin truncate, with 1–3 similar spines. Anterior face of protibia with a very short tarsal groove, tarsi not retractile	Hylesinopsis Eggers

## Systematics

### Hapalogenius

Hagedorn

Fig. 1

Hapalogenius Hagedorn 1912: 352. (Type species: Hapalogenius globosus Hagedorn, monobasic).
Pseudohylesinus Eggers 1920a: 234. (Type species: Pseudohylesinus togonus Eggers, monobasic, preoccupied by Swaine 1917: 11).
Metahylesinus  Eggers 1922: 165. (Type species: Pseudohylesinus togonus Eggers, automatic, replacement name for Pseudohylesinus Eggers nec Swaine).
Pseudophloeotribus Eggers 1933: 18. (Type species: Pseudophloeotribus africanus Eggers, original designation). (The subsequent designation by Schedl 1960: 75 was unnecessary).
Glochicopterus  Schedl 1954: 75. (Type species: Glochicopterus baphiae Schedl, monobasic).
Hapalophloeus Schedl 1966: 363. (Type species: Metahylesinus brincki Schedl, original designation).
Hemihylesinus  Schedl 1967: 224. (Type species: Hemihylesinus endroedyi Schedl, monobasic).Aridiamerus Schedl 1982: 284. (Type species: Aridiamerus angolensis Schedl , monobasic). (Hylesinopsis angolanaWood 1988a: 32 is an unnecessary replacement name.)

Eggers (1927), having compared the types of Phloeotribus fuscipennis (Chapuis, 1869) and Hapalogenius globosus, concluded that the species were identical. Chapuis’ name had priority, but the species did not belong in Phloeotribus and was transferred to Hapalogenius. This conclusion was accepted by Schedl (1963a). Wood and Bright (1992: 94) placed Phloeotribus fuscipennis and its synonym Hapalogenius globosus in Hylesinopsis, overlooking the fact that Hapalogenius has priority, but they also (Wood and Bright 1992: 96) cited Hapalogenius, with its type species, Hapalogenius globosus, as a synonym of Rhopalopselion. As noted above, Alonso-Zarazaga and Lyal (2009) reinstated Hapalogenius as the correct name for the species treated by Wood and Bright (1992) under Hylesinopsis. I consider that Hapalogenius is a valid genus, but that it is distinguished from Rhopalopselion and Hylesinopsis by the characters given in the key above, and with the synonymy given. I have examined the type species of all the genera involved. In addition to the type species listed above, the following 30 nominal species can be assigned to the genus: Hapalogenius acaciae Schedl*, Hapalogenius congonus Schedl, Hapalogenius immaturus Schedl*, Hapalogenius lesnei Eggers, Hapalogenius lonchocarpae Schedl*, Hapalogenius maculatus Schedl*, Hapalogenius occidentalis Schedl*, Hapalogenius primus Schedl, Hapalogenius rufus Schedl*, Hapalogenius senegambiensis Schedl, Hapalogenius subseriatus Schedl*, Hapalogenius suturalis Schedl*, Hylesinopsis kenyae Wood, Hylesinopsis kenyae ugandae Wood*, Hylesinus horridus Eggers, Hylesinus pusillus Gerstaecker, Metahylesinus brincki Schedl*, Metahylesinus dimorphus Schedl, Metahylesinus hispidus Eggers, Metahylesinus orientalis Eggers*, Metahylesinus quadrituberculatus Schedl, Metahylesinus striatus Schedl*, Metahylesinus sulcatus Eggers*, Pseudophloeotribus africanus Eggers*, Pseudophloeotribus oblongus Eggers*, Pseudophloeotribus rhodesianus Eggers*, Pseudophloeotribus seriatus Eggers, Pseudophloeotribus squamosus Eggers*, Pseudophloeotribus variegatus Eggers*, Rhopalopselion atakorae Schedl* (* - type(s) examined). The genus clearly belongs in the tribe Hylesinini *sensu* Wood (1986a), but this tribe seems likely to be paraphyletic (e.g. Farrell et al. 2001, Jordal et al. 2008, McKenna et al. 2009), and may need to be redefined.

### New synonymy in Hapalogenius

#### Hapalogenius africanus

(Eggers)

Pseudophloeotribus africanus Eggers 1933: 19.
Rhopalopselion africanus  (Eggers): Schedl 1951: 1104.
Metahylesinus africanus  (Eggers): Schedl 1960: 76.
Hylesinopsis africanus  (Eggers): Wood 1986: 39.
Hapalogenius africanus  (Eggers): Alonso-Zarazaga and Lyal 2009: 69.
Hapalogenius lesnei Eggers 1943: 73, **syn. n.**
Rhopalopselion lesnei  (Eggers): Wood and Bright 1992: 97.
Metahylesinus brincki Schedl 1957b: 323, **syn. n.**
Glochicopterus brincki  (Schedl): Schedl 1963b: 262.
Hapalophloeus brincki  (Schedl): Schedl 1966: 363.
Hylesinopsis brincki  (Schedl): Wood 1984: 225; Wood and Bright 1992: 93.

I have examined the female holotype of Pseudophloeotribus africanus (NHML) from Zambia, and other specimens from Zimbabwe (NHML), and compared them with syntypes of Hapalogenius lesnei (NMW), and with specimens of Metahylesinus brincki from Namibia which had earlier been compared with syntypes of this species in ZMLU. I have also examined specimens from Angola, Botswana, Namibia, and South Africa (NICP, TMP, RAB). Only a single species is represented, which varies in length from 1.5–2.2 mm, and in size-related characters, such as the number of teeth (6 or 8) on the anterior margin of the pronotum, and the detailed arrangement of the setae on the elytra.

It may be noted that Eggers (1933) described the genus Pseudophloeotribus as having seven funicular segments. The genus Hapalophloeus was separated from Metahylesinus because its type species (Metahylesinus brincki) had only six funicular segments (Schedl 1966). I can confirm that the latter figure is correct. A seventh segment appears to have become fused to the base of the antennal club. The number of funicular segments is normally constant within a species in the Hylesinini.

Hapalogenius africanus is quite widely distributed in southern Africa. In addition to the distribution given by Wood and Bright (1992), it is known from Angola, Botswana and Mozambique. However, no host plants have been recorded. Specimen labels indicate that the species has been collected mostly at light.

#### Hapalogenius fuscipennis

(Chapuis)

Phloeotribus fuscipennis Chapuis 1869: 44.
Hapalogenius fuscipennis  (Chapuis): Eggers 1927: 196.
Hylesinopsis fuscipennis  (Chapuis): Wood and Bright 1992: 94.
Hapalogenius globosus Hagedorn 1912: 352; Eggers 1927: 196 (Synonymy).
Hapalogenius bimaculatus Eggers 1933: 22, **syn. n.**

Eggers (1933) distinguished his new species Hapalogenius bimaculatus from Hapalogenius fuscipennis by the presence of two flecks of dark setae on the posterior third of the elytra among the pale setae of the remainder of the elytra. I have compared the holotype of Hapalogenius bimaculatus (NHML) with a series of specimens of Hapalogenius fuscipennis from South Africa (NCIP, TMP). The series includes specimens in which the elytral setae are wholly pale, intermediates resembling Hapalogenius bimaculatus, and specimens in which the setae are almost entirely dark. In the absence of any other distinguishing characters, I conclude that Hapalogenius bimaculatus is a synonym of Hapalogenius fuscipennis. The shape of the median row of setae on the elytral interstriae varies from almost circular to somewhat elongate and truncate in different individuals. The species is known only from South Africa and Mozambique. Wood and Bright (1992) give Zimbabwe, but this appears to be in error for Mozambique. The only hosts recorded are Millettia grandis (‘Umzimbiti’ of Hagedorn 1912) (Leguminosae), and an unidentified tree ‘sandalo’. Hagedorn (1912) briefly described and illustrated the gallery system under bark.

#### Hapalogenius oblongus

(Eggers)

Pseudophloeotribus oblongus  Eggers 1935: 299.
Metahylesinus oblongus  (Eggers): Schedl 1960: 80.
Hylesinopsis oblongus  (Eggers): Wood and Bright 1992: 95.
Hapalogenius oblongus  (Eggers): Alonso-Zarazaga and Lyal 2009: 69.
Metahylesinus striatus  Schedl 1957: 865, **syn. n.**

The holotypes of both *oblongus* and *striatus* are in NHML. They have been directly compared, and I consider that they represent a single species, with minor variation in the density of the vestiture. I have also examined a series collected by Dr. B. Valentine in Kenya, and compared them with the holotype of Hapalogenius oblongus. The species is recorded from Kenya, Uganda and Tanzania, and from the host tree genera: Acacia (Leguminosae), Croton (Euphorbiaceae) and Eucalyptus (Myrtaceae). This suggests that it is a polyphagous species.

#### Phrixosoma nigra

(Eggers)

Bothryperus niger Eggers 1933: 21
Phrixosoma nigra  (Eggers): Wood and Bright 1992: 190
Hapalogenius niger Schedl 1952: 7, **syn. n.**
Metahylesinus niger  (Schedl): Schedl 1960: 79

I have examined the holotype of Phrixosoma nigra (NHML), and the two syntypes of Hapalogenius niger and a further specimen standing under this name in the Schedl collection (NMW). Both species were described from Uganda, from the same host species (Harungana madagascariensis (Clusiaceae)), and are clearly synonymous. Schedl (1952) appears not to have noticed that the eyes of his species are bipartite, and that the antennal club is asymmetrical with a partly septate first segment – characteristics of Phrixosoma and not of Hapalogenius. Schedl (1963a) briefly describes the biology of the species, and illustrates the gallery system (as Bothryperus niger).

#### Hylesinopsis

Eggers stat. res.

Fig. 2

Hylesinopsis Eggers 1920b: 40. (Type species: Hylesinopsis dubia Eggers, monobasic).
Trypographus  Schedl 1950: 213. (Type species: Trypographus joveri Schedl, monobasic).
Chilodendron  Schedl 1953: 74. (Type species: Chilodendron planicolle Schedl, monobasic).

These genera share the type of eye, antenna and protibia given in the key above. Trypographus and Chilodendron were synonymised with Hylesinopsis by Wood (1983). Wood and Bright (1992: 92) give Chilodendron as a synonym of Hylesinopsis, but its type species, Chilodendron planicolle, is also listed on page 118 under the genus Xylechinus Chapuis, with a note that the transfer of the species was to be published by Wood in a paper in press in 1992. However, this paper was apparently never published. Alonso-Zarazaga and Lyal (2009), apparently basing their decision on the notes in Wood and Bright (1992), synonymise Chilodendron with Xylechinus. I have examined a syntype of Chilodendron planicolle (NMW), and find that the synonymy given by Alonso-Zarazaga and Lyal (2009) appears to be excluded by the 6-segmented funicle (always 5-segmented in Xylechinus), entire eye (always emarginate in Xylechinus), fore tibia without socketed teeth, and plumose metepisternal setae (scalelike in Xylechinus), even though the pronotum lacks asperities (as in some Xylechinus) (Wood 1982, 1986). Until further detailed studies are made of the species here included in Hylesinopsis, I prefer to leave Chilodendron, and its single included species, as a synonym of that genus.

The lack of close relationship of this genus to Hapalogenius, based on morphology, seems to be corroborated by some analyses based on molecular data. In the phylogenetic tree of Farrell et al. (2001: Fig. 6), the single species of Hylesinopsis studied (Hylesinopsis dubia) is widely separated from the two species of Hapalogenius included (Hylesinopsis oblonga, Hylesinopsis seriata) (both labelled as Hylesinopsis sp. in Farrell et al. 2001). In the phylogenetic tree of McKenna et al. (2009: Fig. 2), the same species of Hylesinopsis (Hylesinopsis dubia) (McKenna, pers. comm. 2009) is widely separated from the two genera (Alniphagus, Hylesinus) currently included in the tribe Hylesinini. In both cases, Hylesinopsis seems to be more closely related to genera included in the subfamily Scolytinae *sensu* Wood by Wood (1986) and Wood and Bright (1992). One phylogenetic tree (Jordal et al. 2008, Fig. 4) suggests a closer relationship between Hylesinopsis dubia, Hapalogenius seriata, and Hylesinus varius (F.), but in other analyses the relationship between these species is unresolved (Jordal et al. 2008). The tribal classification of the Scolytinae *sensu* Alonso-Zarazaga and Lyal needs revision (e.g. Jordal et al. 2008, Alonso-Zarazaga and Lyal 2009), and no attempt to place Hylesinopsis in an existing tribe is made here.

In addition to the type species, Hylesinopsis dubia, and the type species of Trypographus (Trypographus joveri),the following nominal species must be removed from Hapalogenius to which they were transferred by Alonso-Zarazaga and Lyal (2009) and returned to Hylesinopsis in which they are listed by Wood and Bright (1992): Chilodendron saudiarabiae Schedl, Hylesinopsis angolensis Schedl, Hylesinopsis arabiae Schedl, Kissophagus alluaudi Lepesme, Kissophagus confusus Eggers*, Kissophagus fasciatus Hagedorn*, Kissophagus ficus Schedl*, Kissophagus granulatus Lepesme, Kissophagus pauliani Lepesme, Kissophagus punctatus Eggers, Trypographus ater Nunberg, Trypographus decellei Nunberg, Trypographus emarginatus Nunberg, Trypographus hirsutus Schedl*. (* - type(s) examined). In addition, the following species belongs to the genus: Cryphalus leprosulus Browne* (see below). References to all these species can be found in Wood and Bright (1992).

The species are normally associated with trees of the family Moraceae (Ficus, Morus, Bosqueia, Treculia). There are only three records from other families, one each from Anacardiaceae, Meliaceae and Rosaceae. This narrow host range contrasts with the wide host range of Hapalogenius and Rhopalopselion.

On the basis of the limited distributional data available, nearly 50% (8 ex 17) of the species appear to be confined to montane habitats above 1500m. This includes the following species: Hylesinopsis alluaudi, Hylesinopsis confusa, Hylesinopsis emarginata, Hylesinopsis fasciata, Hylesinopsis granulata, Hylesinopsis pauliani, Hylesinopsis punctata, Hylesinopsis saudiarabiae. Eight species appear to be more lowland species: Hylesinopsis angolensis, Hylesinopsis arabiae, Hylesinopsis atra, Hylesinopsis decellei, Hylesinopsis dubia, Hylesinopsis ficus, Hylesinopsis joveri, Hylesinopsis leprosula. Hylesinopsis planicolle was described from Mt. d’Ambre in Madagascar, but no altitude is given.

### Taxonomic changes in Hylesinopsis

#### Hylesinopsis fasciata

(Hagedorn)

Kissophagus fasciatus Hagedorn, 1909: 737.
Hylesinopsis fasciatus  (Hagedorn): Wood and Bright 1992: 94.
Chilodendron fasciatus  (Hagedorn): Schedl 1963b: 261.
Kissophagus punctatus Eggers 1932: 28, **syn. n.**

I have compared two specimens of Kissophagus punctatus (NMW), which had been compared with the damaged holotype by Eggers and Schedl respectively, with a series of specimens of Hylesinopsis fasciata in my own collection from Tanzania and Nigeria. The latter had earlier been compared to a syntype of that species, and other specimens from East Africa in NHML. Eggers (1932) distinguished the two species by the more elongate shape, stronger shine, more distinct puncturation, and the presence of granules on the basal part of the elytra. Comparisons suggest that Kissophagus punctatus lies at one end of the range of variation found in Hylesinopsis fasciata. The small differences noted by Eggers (1932) are insufficient to separate Kissophagus punctatus as a separate species, and the latter is, therefore, placed in synonymy. Wood and Bright (1992) cite a holotype for Hylesinopsis fasciata. However, Hagedorn described the species from “compluria specimina”, indicating that he had a series of syntypes before him.

#### Hylesinopsis leprosula

(Browne) comb. n.

Cryphalus leprosulus Browne 1980: 774.

I have examined the holotype (MRAC), and twenty-one paratypes (MRAC, NHML). It is not clear why Browne (1980) assigned this species to the genus Cryphalus Erichson. Such an assignment within the tribe Cryphalini is ruled out by the six–segmented funicle, the elongate eyes, the lack of a visible scutellum, the raised and crenulate basal margin of the elytra, and other characters. The species is here removed from Cryphalus and transferred to Hylesinopsis.

#### Rhopalopselion

Hagedorn

Fig. 3 

When the species described in Hapalogenius are omitted, the remaining eleven species included in Rhopalopselion in Wood and Bright (1992) form a cluster of closely related species distinguished by the quadrate pronotum with strong asperities at the antero-lateral corners, and the large quadrate scutellum. The apical visible sternite has a triangular, median projection. The beetles are strongly built, black in colour, and 2.5–4.5 mm long. All those with known habits are xylophagous (Schedl 1960, Browne 1963). Like Hapalogenius, the genus belongs in the tribe Hylesinini *sensu* Wood (1986a)

In addition to the type species, Rhopalopselion bituberculatum Hagedorn*, I consider the following species to belong in the genus: Rhopalopselion atrum Eggers, Rhopalopselion confusum Eggers, Rhopalopselion conjungens Schedl*, Rhopalopselion dentatum Nunberg*, Rhopalopselion grande Schedl, Rhopalopselion immune Eggers*, Rhopalopselion intermedium Schedl, Rhopalopselion nitidum Schedl, Rhopalopselion orientale Schedl*, Rhopalopselion thompsoni Schedl*. (* - type(s) examined). The remaining species listed under the genus by Wood and Bright (1992) belong in the genus Hapalogenius (see above) in which almost all were originally described. It may be noted here that the holotype of Rhopalopselion bituberculatum is in DEI and not MNB as stated by Wood and Bright (1992).

### New records of Hapalogenius and Hylesinopsis

The following new records extend the known geographical distribution of the species.

Hapalogenius atakorae (Schedl). GHANA: Northern Reg., Nakpanduri escarp., 10°38'N; 0°12'W, 19.vi.1971, under tree bark (*Endrödy-Younga*) (37exx.) (TMP, RAB).

Hapalogenius pusillus (Gerstaecker). SOUTH AFRICA, West Cape, Knysna, Gouna, 6.xi.2006, ex Virgilia oroboides (*B. Jordal*) (4exx.) (RAB) (Further specimens in B.Jordal’s collection).

Hapalogenius sulcatus (Eggers). NAMIBIA: East Caprivi, Katima, Mulilo, 17°29'S; 24°17'E, 3–8.iii.1992 (*M. Uhlig*) (1 ex.); Kavango, Kaudom Camp, 18°21'S; 20°43'E, 22–25.ii.1992, lux (*M. Uhlig*) (1 ex.); Kavango, Mahango Game Res., 20.i.1993 (*F. Koch*) (ZMB); SOUTH AFRICA: E.Transvaal, Berlin, 300m below, 25°33'S; 30°43'E, 4.ii.1987, UV light (E-Y:2416) (1 ex.); N.Transvaal, Entabeni – L.Trich.,c. 23°05'S; 30°12'E, airplankton (E-Y:1138) (1 ex.); Tvl., Nelshoogte, gallery for. below St., 25°51'S; 30°53'E, 4.xii.1987, UV light (E-Y:2354) (1ex.); Tvl., Nelspruit Nat.Res., dry valley, 25°29'S; 30°55'E, 8.ii.1987, UV light, top valley (E-Y:2432) (4 exx.) (all coll. *Endrödy-Younga*) (TMP, RAB); ZIMBABWE: Chipinga, 1.ii.1990 (*C. R. Owen*) (1 ex.) (TMP); Kyle Recr. Park at Lake Mutirikwi, 20°13'S; 31°00'E, 1–5.xii.1993, lux (*M. Uhlig*) (1 ex.) (ZMB).

Hylesinopsis dubia Eggers. GUINEA: Seredou, 4.iv.1975, lux (*Zott*) (1 ex.) (ZMB).

Hylesinopsis fasciata (Hagedorn). SOUTH AFRICA : S.Natal, Weza, Bangeni Forest, 30°38'S; 29°39'E, 21.xi.1989, beating in forest (*Endrödy & Klimaszew*) (E-Y:2708) (1 ex.); as previous except: 30°32'S; 29°41'E, 23.xi.1989 (E-Y: 2716) (1 ex.) (TMP).

## Supplementary Material

XML Treatment for Hapalogenius

XML Treatment for Hapalogenius africanus

XML Treatment for Hapalogenius fuscipennis

XML Treatment for Hapalogenius oblongus

XML Treatment for Phrixosoma nigra

XML Treatment for Hylesinopsis

XML Treatment for Hylesinopsis fasciata

XML Treatment for Hylesinopsis leprosula

XML Treatment for Rhopalopselion
